# Environmental Change in the Agro-Pastoral Transitional Zone, Northern China: Patterns, Drivers, and Implications

**DOI:** 10.3390/ijerph13020165

**Published:** 2016-01-28

**Authors:** Chong Jiang, Fei Wang

**Affiliations:** 1 Institute of Soil and Water Conservation, Northwest A&F University, Yangling 712100, China; 2 College of Global Change and Earth System Science, Beijing Normal University, Beijing 100875, China; 3 Institute of Soil and Water Conservation, Chinese Academy of Sciences & Ministry of Water Resources, Yangling 712100, Shaanxi Province, China

**Keywords:** agro–pastoral transitional zone, water resource, water environment, atmospheric environment, vegetation activity

## Abstract

Chengde city is located in the agro–pastoral transitional zone in northern China near the capital city of Beijing, which has experienced large-scale ecological construction in the past three decades. This study quantitatively assessed the environmental changes in Chengde through observation records of water resources, water environment, atmospheric environment, and vegetation activity and investigated the possible causes. From the late 1950s to 2002, the streamflow presented a downward trend induced by climate variability and human activities, with contribution ratios of 33.2% and 66.8%, respectively. During 2001–2012, the days of levels I and II air quality presented clear upward trends. Moreover, the air pollutant concentration was relatively low compared with that in the adjacent areas, which means the air quality has improved more than that in the neighboring areas. The water quality, which deteriorated during 1993–2000, began to improve in 2002. The air and water quality changes were closely related to pollutant emissions induced by anthropogenic activities. During 1982–2012, the vegetation in the southeastern and central regions presented restoration trends, whereas that in the northwestern area showed degradation trends. The pixels with obvious degradation trends correlated significantly with annual mean temperature and annual precipitation. Ecological engineering also played a positive role in vegetation restoration. This analysis can be beneficial to environment managers in the active response and adaptation to the possible effects of future climate change, population growth, and industrial development and can be used to ensure sustainable development and environmental safety.

## 1. Introduction

The fifth assessment report of the Intergovernmental Panel on Climate Change (IPCC) showed that the air temperature presented a global warming trend during the past half-century [1], with the fastest warming rate in the mid-latitudes of the Northern Hemisphere [2]. Temperature warming accelerates global and regional water cycles, which may induce extreme climatic and hydrological events and lead to redistributions of water resources and ecosystems at various scales [3]. Moreover, the human population has been continuously increasing, with an expected addition of 2 to 4 billion people by 2050 [4,5]. Anthropogenic activities have thus far imposed large environmental impacts on the earth [6]. Environmental degradation induced by the ever-increasing extent and intensity of anthropogenic activities has been and will continue to be the most perplexing challenges in sustainable development [7]. Under this background, severe environmental problems such as soil erosion, sand storms, desertification, and wildlife habitat losses have increased at the global and regional scales [1,2]. As the largest developing country, China’s economic status continues to maintain rapid growth, and therefore has suffered from severe environmental degradation during the past three decades [8,9]. The estimated economic loss associated with environmental degradation, such as environmental pollution, resource exhaustion, and ecological degradation, has amounted to over 13% of the gross domestic product (GDP) [10]. 

To improve the environmental conditions in China, ecological remediation and conservation measures have been widely recognized and implemented as important and effective measures to fight environmental degradation [11,12,13]. Given the widely implemented ecological restoration projects, the effectiveness of these conservation and restoration efforts has become a hot issue that has practical implications [11,12]. There is an increasing need to quantitatively evaluate the effectiveness of ecological conservation and restoration, particularly on large spatial scales, to improve the decision support for land management and ecological restoration planning and implementation [14]. Moreover, determining the exact manner in which global climate change and ecological conservation affect the eco-environmental health has been an important research field for some international projects such as the IPCC, the Future Earth–International Council for Scientific Unions (ICSU), the International Geosphere–Biosphere Program (IGBP), and other international organizations and projects.

Chengde city is located in the agro–pastoral transitional zone in northern China near the capital city of Beijing. During the past three decades, the Chinese government has initiated several large-scale ecological restoration programs in Chengde and its adjacent areas, including the Beijing–Tianjin Sand Source Control Project, the Three-North Shelterbelt Project, and the Grain for Green Project [15,16,17,18]. Previous research includes systematic and in-depth studies on environmental changes in this area, such as studies on carbon cycle [17], dust weather [18], and vegetation cover change [18]. However, previous research has focused mainly on the environmental changes after implementation of ecological projects. That is, existing research focuses mainly on the dynamic monitoring and effectiveness assessments of single and multiple ecological projects; few studies have investigated the drivers, causes, and implications of environmental change. In addition, existing studies are updated only to the early 2000s; relatively limited information is available on environmental changes earlier than 2000, and very few studies include more than three decades of data. Some frequently occurring regional environment phenomena such as water pollution, air pollution, water resource shortages (streamflow reduction), and vegetation degradation and restoration, have not been adequately investigated or understood. Therefore, it is necessary to conduct an in-depth investigation on regional environmental changes and the responses to anthropogenic activities and climate variability, which will play an important role in future sustainable development.

Therefore, the objectives of this study were: (1) to quantitatively assess the environmental changes in Chengde city since the late 1950s through observation records of water resources, water environment, atmospheric environment, and vegetation activity; (2) to investigate the possible causes for regional environmental changes; and (3) to discuss the practical and policy implications of environmental changes and to provide a reference for environmental policy making and planning. The results of this study can be used to examine the following research topics: (1) the coexistence of positive and negative environmental impacts in Chengde city owing to climate variability and anthropogenic activities; (2) the environmental changes most relevant to anthropogenic activities; and (3) the heterogeneity of regional environmental and climatic changes.

## 2. Data and Methods

### 2.1. Overview of the Research Area

**Figure 1:**
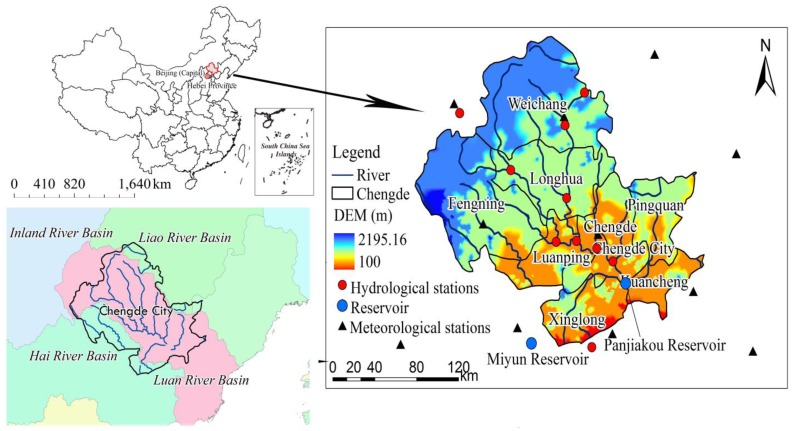
Locations of the study area, hydrological station, and meteorological station in Chengde.

The city of Chengde is located in northern China, bounded by 40°12′–42°37′ N and 115°54′–119°15′ E (Figure 1), and its total area is 39,808.9 km^2^, accounting for 21.2% of the entire area of Hebei Province. The topography is characterized by low mountains and hilly landscapes, with elevation varying from 100.0 m to 2195.2 m above sea level. The forest and grassland in Chengde account for 43.4% and 40.0% of total area in Hebei Province, respectively. The region has a semi-humid continental monsoon climate with a mean annual temperature of 8.4 °C. Precipitation is temporally variable, with more than 82% of the entire year’s rainfall occurring in June–September; the mean annual precipitation is 533.1 mm. The three major river systems in Chengde include the Luan River (28,878.4 km^2^), Chao River (6776.7 km^2^), and Liao River (4153.8 km^2^), and the average water yield is about 37.6 × 10^8^ m^3^. These rivers are an important water source for the Panjiakou and Miyun reservoirs, which are the two largest drinking-water reservoirs for Beijing and its adjacent areas. As an important ecological barrier for Beijing, it is close to the Inner Mongolia autonomous region, which is the main source of dust in northern China. To improve the ecological conditions, the Chinese government adopted a series of large-scale ecological restoration programs [15,16,17,18]. This area experienced intensive urbanization and industrial development in the past three decades, which induced obvious land use change [17,18]. Moreover, the multi-source environmental monitoring data is relatively complete in this region. For these reasons, Chengde is an ideal area for environment change assessment. 

### 2.2. Data and Processing

The data sources and detailed information of datasets used in this study are presented in Table 1 and Supplementary Tables S1–S4. 

**Table 1:** Datasets used in environmental change assessments including data types, dataset names, specific information, and sources.

Data Types	Dataset Name and Specific Information	Source
Basic geographic information	Administrative map (1:4 million-scale)	The National Geomatics Center of China [19]
	The Shuttle Radar Topography Mission (SRTM) digital elevation model (DEM) (resolution:90 m)	Consultative Group on International Agricultural Research-Consortium for Spatial Information (CGIAR-CSI) [20]
	River and lake system atlas (1:1 million-scale)	The National Geomatics Center of China [19]
Hydrological and meteorological data	Annual water discharge and sediment load in six hydrological stations from late 1950s to 2002	[21,22]
	Daily meteorological data in twelve meteorological stations during 1956–2011	National Meteorological Data Sharing Service System [23]
Remote sensing image	GIMMS NDVI3g dataset from NOAA’s AVHRR sensors during 1982 to 2012 (resolution: 8 km × 8 km)	National Aeronautics and Space Administration (NASA) [24]
	Land use maps in 1985, 2000, and 2010 (1:0.1 million-scale)	Data Center for Resources and Environmental Sciences, Chinese Academy of Sciences (RESDC) (http://www.resdc.cn) [25]
Environmental monitoring data	Monthly water quality record of 17 monitoring stations from late 1980s to early 2000s Annual air and water quality records of Chengde city and Hebei Province during 2000–2012	[22]
		[26,27]
The social and economic data	Statistical yearbook for Chengde city	Bureau of Statistics in Hebei Province, China

Excluding a few stations having large amounts of missing data, the daily meteorological data were collected from meteorological stations in Chengde and its neighboring areas. The spatial distribution of meteorological stations is shown in Figure 1. Complete records for almost all of the climatic factors from 1956 to 2011 were obtained; the inverse distance weighted technique was used to interpolate missing data. Records of water discharge and sediment load from the late 1950s to 2002 were obtained from six hydrological stations chosen within Chengde and its neighboring basins [21,22]. The observational records of atmospheric and water pollutants were obtained from the Environmental Monitoring Station of Hebei Province [22,26]. The land use maps examined in this study were obtained from the Data Center for Resources and Environmental Sciences, Chinese Academy of Sciences (RESDC) [25]. Landsat Thematic Mapper (TM), Enhanced Thematic Mapper (ETM), and China–Brazil Earth Resources Satellite (CBERS) images were collected to interpret the land cover maps of 1985, 2000, and 2010 [28,29,30]. Prior to interpretation, the remote sensing images were subjected to geometric correction by using topographic maps at a scale of 1:0.1 million. The land cover types were identified on the basis on the spectral reflectance and structure of objects [28,29,30]. The 27 land cover subtypes identified were further grouped into seven aggregate types including grassland, woodland, residential areas, farmland, desert, water bodies, and unused land, with a classification accuracy of more than 90% [28,29,30]. The third-generation Global Inventor Modeling and Mapping Studies Normalized Difference Vegetation Index (GIMMS NDVI 3g) products from the Advanced Very High Resolution Radiometer (AVHRR) sensors of the National Oceanic and Atmospheric Administration (NOAA) during 1982 to 2012 were used to investigate the changes in vegetation activity. The GIMMS NDVI 3g dataset, with a temporal resolution of two weeks, is an updated version of the previous GIMMS NDVI [31]. The maximum value composites (MVC) [32] were used to eliminate cloud and aerosol effects and were then aggregated into two-week composites. This dataset, which is widely applied is global and regional studies, was corrected to calibrate the view geometry [32,33].

### 2.3. Methods

#### 2.3.1. Indicator System for Environmental Change Assessment

To quantitatively assess the regional environmental changes in Chengde, we established a framework in which several indicators were chosen on the basis of observation records and remote sensing image. The environmental changes include water environment, water resources, atmospheric environment, and vegetation activity. The specific indicators and assessment methods are given in Table 2. Chengde experienced intensive human activities and ecological construction in the past three decades, which consumed large amounts of water resources and induced water pollution. Because this area is semi-arid, the water resources and water environment were considered first in the environmental assessment. In consideration of the data availability, type of pollutant, representativeness, and comprehensiveness of indictors, the selected indicators for the water environment assessment include chemical oxygen demand (COD), biochemical oxygen demand (BOD), NH_3_-H, MnO^4−^, volatile phenols, and total hardness. In terms of the atmospheric environment, dust haze and fine particulate matter (PM_2.5_) events were prominent in the recent decades; thus, both of these indices were included. In addition to this, the emission amounts of COD and NH_3_-H is included in attribution. The vegetation activity was reflected by the NDVI derived from remote sensing images, which is a widely used indicator in vegetation growth monitoring at global and regional scales.

**Table 2:** Indicator system for environmental change assessment.

Assessment Projects	Indicators	Unit	Assessment Method
Water resources	Streamflow	m^3^	Observational record
Water environment	Sediment load	10^4^ t	Observational record
	Pollutant concentration: COD, NH_3_-H, MnO^4−^, volatile phenol	mg/L	
	Pollutant emission: COD, NH_3_-H	t/km^2^	
	Other indicators: BOD, total hardness	mg/L	
Vegetation activity	NDVI	Dimensionless	GIMMS NDVI3g dataset
Atmospheric environment	Pollutant concentration: PM_2.5_	μg/m^3^	Observational record
	Pollutant emission: SO_2_, NO_x_	mg/L	
	Dusthaze days	day	

#### 2.3.2. Mann–Kendall Trend Detection

The Mann–Kendall test, one of the non-parametric tests widely used to detect change trends of hydrological and climatic variables in a time series [34,35], is based on the statistic *S*:

(1) $$S=\sum_{i=1}^{n-1}\sum_{j=i+1}^{n}\text{sgn}\left(x_{j}-x_{i}\right)$$

(2) $$\text{sgn}\left(x\right)=\{\begin{array}{ccc} 1, & if & x_{j}-x_{i}>0\\ 0, & if & x_{j}-x_{i}=0\\ -1, & if & x_{j}-x_{i}<0 \end{array}$$

where *x*_i_ and *x*_j_ are two generic sequential data values of the variable, and *n* is the length of the dataset. The null hypothesis implies no trend in the dataset, and the statistic *S* is approximately distributed normally with a mean of zero. For datasets with more than 10 values, the variance associated with the statistic *S* (*Var*(*S*)) can be calculated as:

(3) $$Var\left(S\right)=\frac{n\left(n-1\right)\left(2n+1\right)}{18}$$

The values of *S* and *Var*(*S*) are used to compute the test statistic *Z* as:

(4) $$Z=\{\begin{array}{ccc} \frac{S-1}{\sqrt{Var\left(S\right)}} & if & S>0\\ 0 & if & S=0\\ \frac{S+1}{\sqrt{Var\left(S\right)}} & if & S<0 \end{array}$$

The presence of a statistically significant trend is evaluated by using the *Z* value. A positive (negative) value of *Z* indicates an upward (downward) trend. The statistic *Z* has a normal distribution. The null hypothesis can be rejected at the significance level of α if |*Z*| ≥ *Z*_1 −_ α_/2_, where *Z*_1 −_ α_/2_ is obtained from the standard normal cumulative distribution tables [36].

#### 2.3.3. Mann–Kendall Change-Point Analysis

To conduct change-point analysis of the hydrological and climatic variables in time series, the non-parametric Mann–Kendall test was employed. The test statistic *S_k_* is defined as:

(5) $$S_{k}=\sum_{i=1}^{k}\sum_{j=1}^{i-1}\alpha_{ij}\left(k=2,3,4,\ldots ,n\right)$$

(6) $$\alpha_{ij}=\{\begin{array}{cc} \begin{array}{cc} 1 & x_{j}>x_{i}\\ 0 & x_{j}\leq x_{i} \end{array} & 1\leq j\leq i \end{array}$$

Then, the definition of the statistic index *UF* is calculated as:

(7) $$UF=\frac{S_{k}-E\left(S_{k}\right)}{\sqrt{Var\left(Var\left(S_{k}\right)\right)}}\begin{array}{cc} & k=1,2,3,\ldots ,n \end{array}$$

(8) $$E\left(Sk\right)=\frac{k\left(k-1\right)}{4}$$

(9) $$Var\left(S_{k}\right)=\frac{k\left(k-1\right)\left(2k+5\right)}{72}$$

*UF* follows the standard normal distribution, which is the forward statistic sequence. The backward sequence *UB* is calculated by using the same equation but with a reversed series of data.

In the two-sided test, if the null hypothesis is rejected, an increasing (*UF* > 0) or a decreasing (*UF* < 0) trend is indicated. If there is a match point of the two curves and the trend of the series is statistically significant, the match point would be regarded as the change point [37].

#### 2.3.4. Evapotranspiration Estimation with the FAO Penman–Monteith Equation

As recommended by the Food and Agriculture Organization (FAO), the Penman–Monteith equation was modified to better account for specific local conditions [38]. In this study, several parameters were calibrated by using the observed data of each station for estimating the net radiation (*R*_n_). This study adopted the FAO Penman–Monteith method to estimate the daily *E*_0_ (mm^.^d^−1^):

(10) $$E_{0}=\frac{0.408\Delta (R_{n}-G)+r\frac{900}{T+273}U_{2}(e_{s}-e_{a})}{\Delta +r(1+0.34U_{2})}$$

where *E*_0_ is potential evapotranspiration; *R_n_* is the net radiation at the crop surface (MJ·m^−2^·d^−1^), where MJ represents mega joules; *G* is the soil heat flux density (MJ·m^−2^·d^−1^); *T* is the air temperature at a height of 2 m (°C); *U*_2_ is the wind speed at a height of 2  m (m·s^−1^); *e*_s_ is the saturation vapor pressure (kPa); *e*_a_ is the actual vapor pressure (kPa); Δ is the slope of the vapor pressure curve (kPa·°C^−1^); and *γ* is the psychrometric constant (kPa·°C^−1^).

#### 2.3.5. Estimating the Impact of Climate Variability on Streamflow

It can be assumed that a change in mean annual runoff can be determined by using the following expression [39,40]:

(11) $$\Delta Q_{c\text{lim}}\;=\;\beta \Delta P\;+\gamma \Delta E_{0}$$

where Δ*Q_clim_* is the change in streamflow caused by climate variability; Δ*P* and Δ*E*_0_ are changes in precipitation and potential evapotranspiration, respectively; *β* is the sensitivity of streamflow to precipitation; and *γ* is the sensitivity to potential evapotranspiration.

The sensitivity coefficients can be expressed as:

(12) $$\beta =\frac{1+2x+3\omega x}{\left(1+x+\omega x^{2}\right)}$$

(13) $$\gamma =-\frac{1+2\omega x}{\left(1+x+\omega x^{2}\right)}$$

where x is the index of dryness and is equal to *E*_0_/*P*, and *ω* is the plant-available water coefficient, which represents the relative difference in the way plants use soil water for transpiration.

#### 2.3.6. Vegetation Growth Change and Trend Estimation

The vegetation activity can be reflected by the NDVI, and the slope of change trend can be derived by using the least squares method, which is commonly used in trend estimation:

(14) y=a× t+b+ε

where *y* is the annual maximum *NDVI* in year *t*, *a* is the slope of equation (change rate of linear trend), *b* is the intercept of equation, and *ε* is the random error. *t* ranges from 1982 to 2012 (*t* ∈ N); therefore, the sample size of the annual maximum *NDVI* for each pixel in the vegetation change trend estimation is 31. *a* < 0 represents weakness in the vegetation growth. By contrast, *a* > 0 represents strengthening of the vegetation growth. The calculation process was conducted by using MATLAB software with an orthogonal least squares method [41].

#### 2.3.7. Correlation Analysis between Vegetation Cover and Climatic Factor

Pearson’s correlation analysis was performed by using the function of [*r*, *p*] = corr (*X*, *Y*) in MATLAB software, which can simultaneously return both the correlation coefficient and significant test (*p*-value), where *r* and *p* represent the correlation coefficient and significant test result, respectively, and *X* and *Y* are the vegetation cover and climate factor, respectively.

## 3. Results

### 3.1. Climatic Change

**Figure 2:**
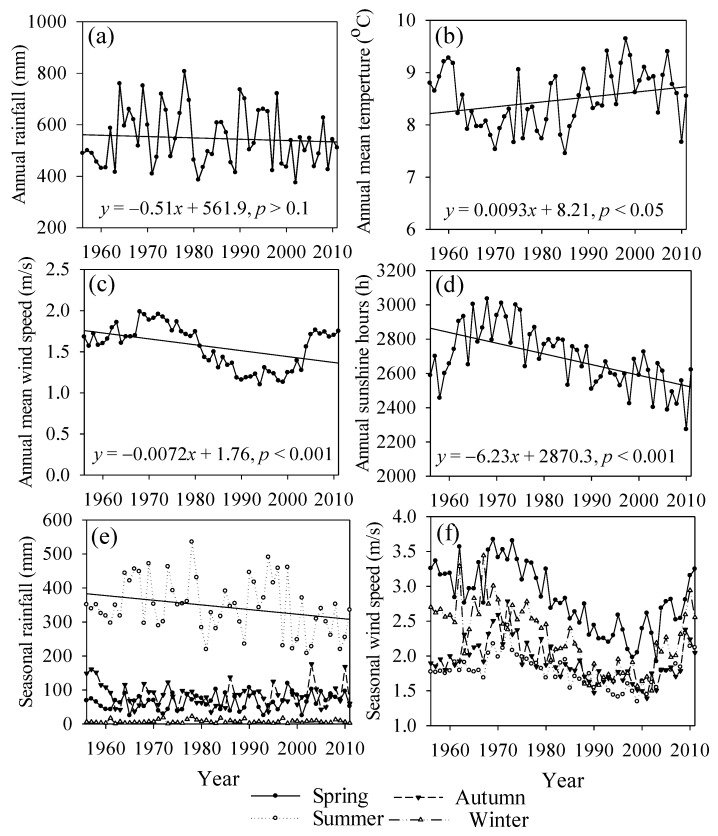
Temporal changes in meteorological factors in Chengde from 1956 to 2011 including (**a**) annual precipitation; (**b**) annual mean temperature; (**c**) annual mean wind speed; (**d**) annual sunshine hours; (**e**) seasonal rainfall; and (**f**) seasonal mean wind speed.

The annual precipitation in Chengde fluctuated dramatically from 1956 to 2011 (Figure 2a) and remained at relatively high levels from 1956 to 1980. The most humid period, in 1970–1980, was followed by the driest period in 1980–1990. Since 2000, the precipitation has presented an upward trend, although it is still lower than that in the 1970s. The increase rate of annual mean temperature in Chengde was 0.09 °C/decade (Figure 2b), which is slightly lower than the global average of 0.12 °C/decade [1]. A significant temperature increase occurred during the mid-1980s; from 1956 to 1985, the temperature increased to a lesser extent. An abrupt change in temperature occurred in 1985, and the increasing rate of 0.17 °C/decade indicates an accelerating trend. The yearly mean wind speed presented significantly decreasing trends on the entire scale of 1956–2011, although an obvious upward trend was shown in 2000 (Figure 2c). The annual sunshine hours presented significantly decreasing trends on the entire scale of 1956–2011 (Figure 2d), which reduced the solar radiation for vegetation growth. 

Because wind and rainfall are the main soil erosion forces and rainfall directly influences intra-annual distribution and inter-annual changes in streamflow, we focused on the rainfall and wind speed changes at the seasonal scale (Figure 2e,f). The rainfall in summer and autumn presented decreasing trends with slopes of −13.7 mm/decade and −0.12 mm/decade, respectively; that in summer reached the *p* = 0.05 significance level. The rainfall in spring and winter showed upward trends but did not reach the significance level. Regarding the seasonal wind speed, the change trends in the four seasons were essentially consistent. The slope in spring was largest at −0.16 m/(s·decade) (*p* < 0.001), which is higher than that at the annual scale. 

### 3.2. Land Use/Land Cover Change 

The land cover in Chengde was grouped into seven aggregated types including grassland, woodland, residential areas, farmland, desert, water bodies, and unused land, as shown in Table 3. The land cover change in the period 2000–2010 was not obvious, and the percentage of area change in multiple land cover types was marginal. In comparison, the main changes in 1985 and 2010 occurred in woodland (−5.0%), farmland (1.6%), and residential areas (1.4%). Rapid urbanization and population growth caused dramatic expansion in residential areas and farmland, which occupied some areas of woodland. Some forests degraded into grassland such as shrubs in addition to desert; thus, the percentage of grassland and desert increased 0.9% and 0.6%, respectively. Owing to ecological restoration and water conservancy projects, the area of water bodies and wetland increased slightly at 0.2%. 

**Table 3:** Area of land use and its change in Chengde during 1985–2010.

Types	Percent of Area and Change (%)					
	1985	2000	2010	1985–2000	2000–2010	1985–2010
Residential areas	2.5	3.8	3.9	1.3	0.2	1.4
Farmland	16.9	18.7	18.5	1.8	−0.1	1.6
Woodland	65.2	60.2	60.2	−5.0	0.0	−5.0
Grassland	13.9	14.8	14.8	0.9	0.0	0.9
Desert	0.0	0.6	0.6	0.6	0.0	0.6
Water bodies and wetland	1.1	1.4	1.3	0.3	0.0	0.2
Unused land	0.4	0.5	0.5	0.1	0.0	0.1

### 3.3. Water Resources Change and Attribution

#### 3.3.1. Trend and Change Point in Annual Streamflow

**Figure 3:**
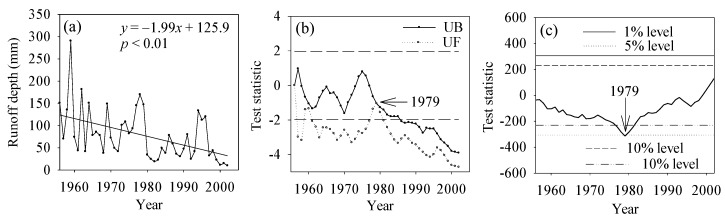
(**a**) Inter-annual variability of annual runoff depth from 1956 to 2002 in Chengde; (**b**) Mann–Kendall change-point detection and (**c**) Pettit change-point detection in the time series of annual streamflow. The annual streamflow of Chengde is the sum of flow recorded by stations in six sub-basins.

**Figure 4:**
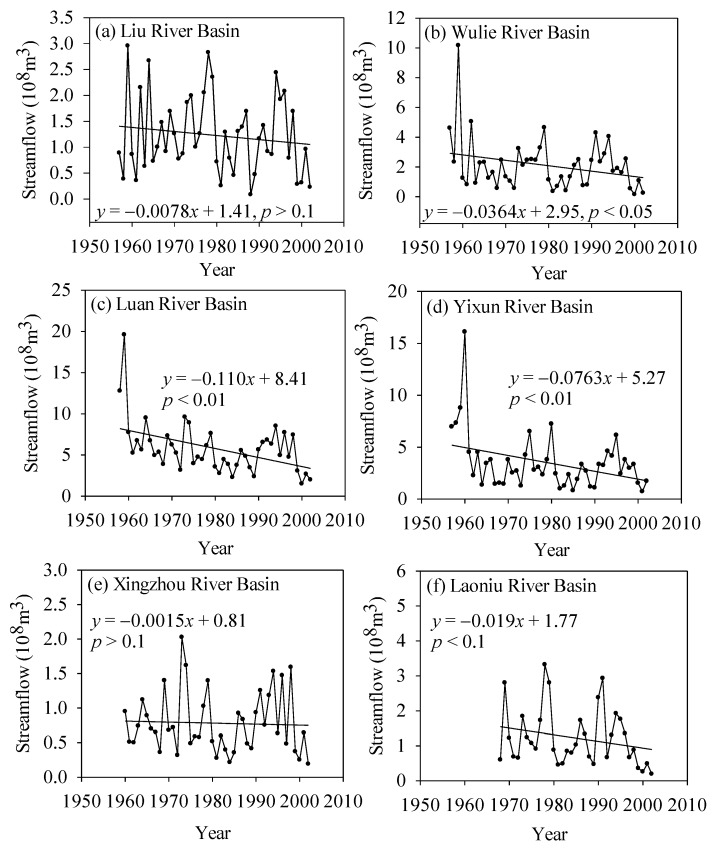
Inter-annual variability of annual streamflow from late 1950s to 2002 in the sub-basins: (**a**) Liying Station in Liu River; (**b**) Chengde Station in Wulie River; (**c**) Sandaohezi Station in Luan River; (**d**) Hanjiaying Station in Yixun River; (**e**) Boluonuo Station in Xingzhou River; and (**f**) Xiabancheng Station in Laoniu River.

Trend analysis is useful for understanding the dynamics and long-term behavior of hydrological and climatic variables. The Mann–Kendall test was applied to the annual streamflow data of period 1956–2002. The *Z* statistic of the runoff depth in Chengde was −3.04, showing a significant downward trend at the 0.01 confidence level (Figure 3a). The streamflow in the six sub-basins presented downward trends during the past several decades, some of which reached the significance level (Figure 4). Figure 3b,c show the Mann–Kendall and Pettitt change-point test [42] of the streamflow data, in which the intersection of the curves indicates an abrupt change in 1979 at the 0.05 significance level. Based on the change point of 1979, the period of the streamflow record was divided into two parts including a reference period of 1956–1979 and a change period of 1980–2002.

The negative UF value also shows the decreasing trend in annual streamflow. It is clear that annual streamflow during the two periods differed significantly, with reduced streamflow occurring in the second period (Figure 3a). The mean annual streamflow in the reference period was 115.6 mm, whereas the value in the change period was only 54.3 mm. These results show a significant reduction of 61.3 mm, or 53.0%, in the change period.

#### 3.3.2. Attribution of Water Resources Change 

Compared with the baseline period of 1956–1979, the annual precipitation decreased 48.9 mm, or 5.37%, in the change period of 1980–2002; however, the potential evapotranspiration showed a slight change of 8.34 mm, or an increase of only 0.81%. Because of the small change in *E*_0_, the effects of climate on streamflow were caused only by the change in precipitation. Therefore, Equation (10) can be simplified as:

(15) $$\Delta Q_{c\text{lim}}=\beta \Delta P$$

In research conducted in China [43] and elsewhere [44,45], the assigned *ω* parameter is 2.0 for high-cover woodland when forest cover is >30%, 1.0 for low-cover woodland when forest cover is <30%; 0.5 for grassland and cropland; 1.0 for shrubland; and 0.1 for buildings and barren land.

In this study, the coverage of land use was determined by land-use maps. By using those of 1985, 2000, and 2010, the mean value of different land-use/cover types was obtained. The sensitivity of streamflow to precipitation (*β*) was calculated as Equation (11), which showed a *β* value of 0.416. Therefore, the change in streamflow caused by climate variability (Δ*Q_clim_*), calculated as Equation (15), is 20.3 mm, accounting for 33.2% (Table 4). According to the Δ*Q* value of 61.3 mm, the change in streamflow induced by human activities (Δ*Q_hum_*) is 40.9, accounting for 66.8%.

**Table 4:** Streamflow impacts induced by climate variability and human activities.

Item	Δ*Q*	Δ*Q_clim_*	Δ*Q_hum_*
Quantity/mm	61.3	20.3	40.9
Contribution rate	100%	33.2%	66.8%

**Figure 5:**
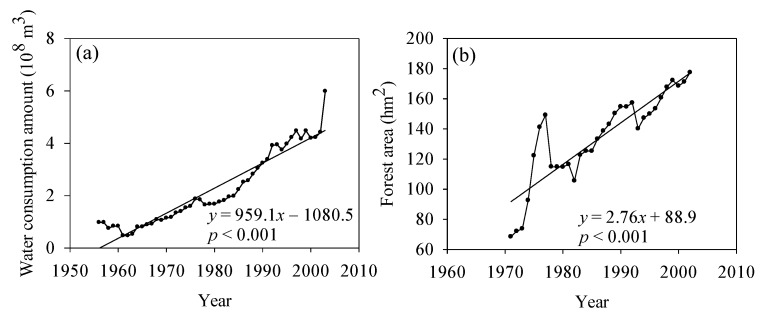
(**a**) Water consumption during 1956–2003 and (**b**) forest area during 1971–2002 in Chengde.

Figure 5a shows that the water consumption increased significantly during the past half century, with a trendline slope of 959.1 × 10^4^ m^3^/a (*p* < 0.001). The dramatic increase in forest area caused by ecological construction also induced ecological water consumption (Figure 5b). However, it should be noted that the ecological water consumption accounted for only a small proportion of total water consumption (figure omitted), at less than 4%, and the domestic water consumption accounted for less than 10%. Industrial water consumption had the largest proportion and was thus the main cause of the streamflow reduction. 

### 3.4. Water Environment Change and Attribution

#### 3.4.1. Water Environment Change 

**Figure 6:**
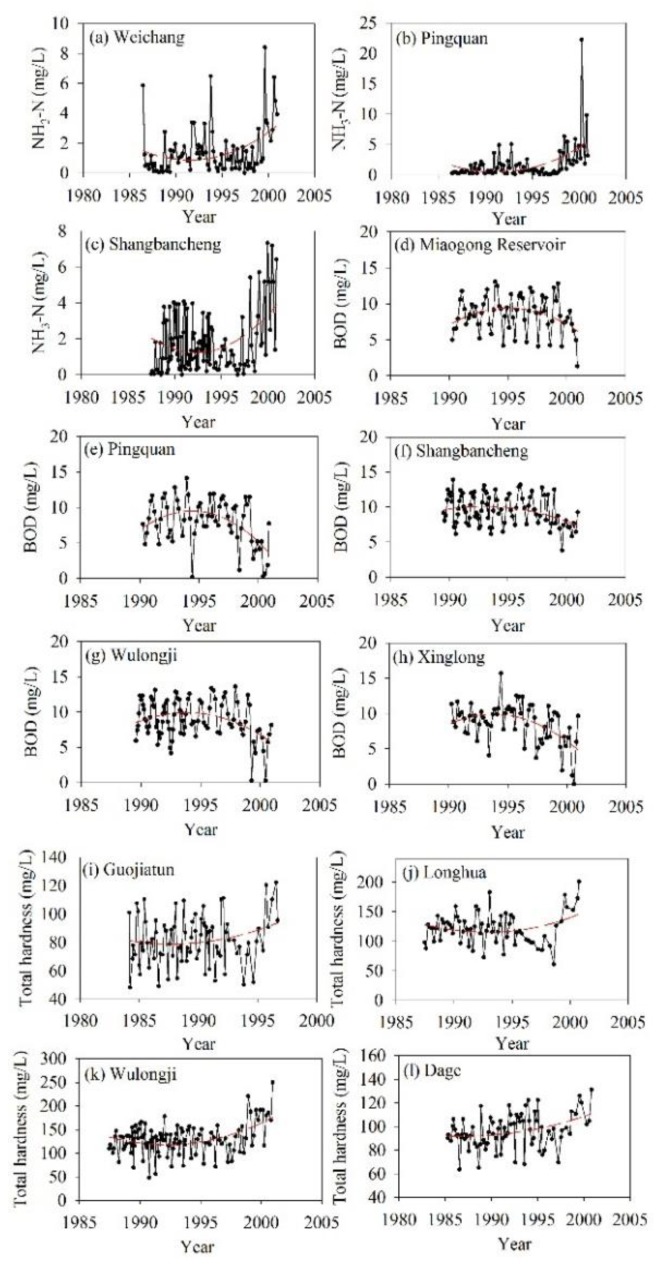
Inter-annual variability of water quality indicators from the late 1980s to 2002 in the sub-basins. (**a**–**c**) indicate NH_3_-N; (**d**–**h**) indicate BOD; and (**i**–**l**) indicate total hardness. Red lines are trendlines fitted by the polynomial.

**Figure 7:**
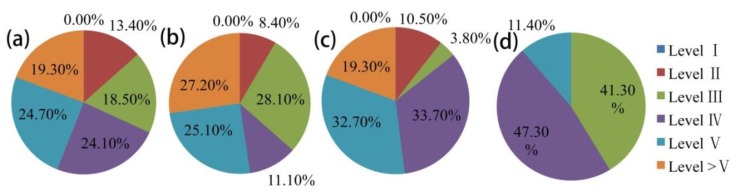
Statistical results of basin area with different levels of water quality in 2000. (**a**–**c**) indicate the water quality of surface water at the annual scale, flood season, and dry season, respectively; (**d**) shows the water quality of groundwater at the annual scale.

**Figure 8:**
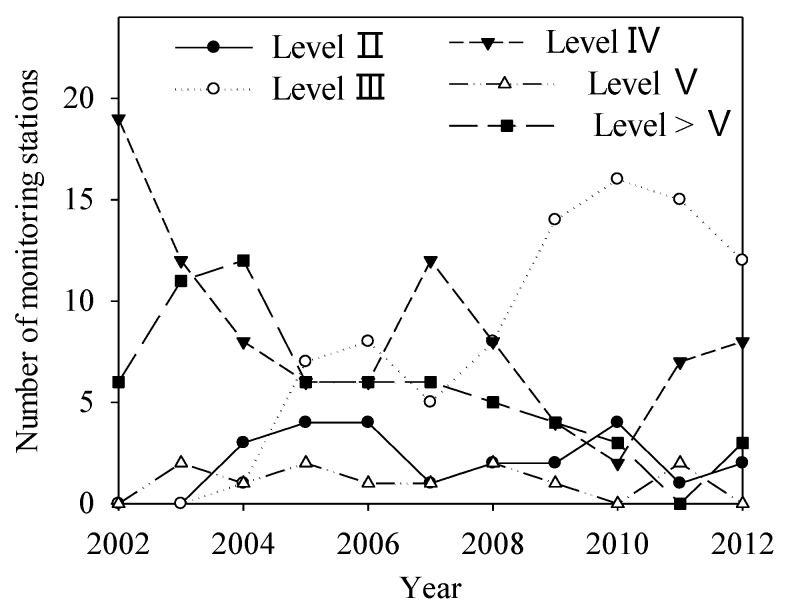
Number of stations with varying levels of water quality during 2002–2012.

Considering the data availability for water quality, we chose only the water quality monitoring data from 14 stations recorded between 1993 and 2000. The statistical results are shown in Table 5. During 1993–2000, the MnO^4−^ and volatile phenol levels at most stations maintained stability. For the total hardness and NH_3_-N, stations showing upward trends accounted for more than 50%; BOD, which is a sign of water quality deterioration, showed a downward trend at 12 stations, or 70.6%. The temporal variation of specific indicators at representative stations is shown in Figure 6. The time series analysis indicated that the total hardness at Guojiatun, Longhua, Wulongji, and Dage stations began to increase around 1996 and 1997; NH_3_-N at Weichang, Pingquan, and Shangbancheng stations showed a clear upward trend; and BOD at Miaogong Reservoir and Pingquan, Shangbancheng, Wulongji, and Xinglong stations presented clear downward trends. According to the national quality standard for surface water [46], in 2000, more 60% of the surface water in the basin area was of quality lower than level III in flood and dry seasons and on the annual scale, which prohibits its use as a drinking water source (Figure 7a–c). In addition, the groundwater quality in more than 50% of area was lower than level III on the annual scale (Figure 7d). However, the water quality began to improve in 2002 (Figure 8). The monitoring stations with water quality equal to levels III and II increased significantly, and those with water quality lower than level III reduced significantly.

**Table 5:** Temporal variation of surface water quality indicators during 1993–2000 in Chengde.

Basin	Station	Total Hardness	MnO^4−^	NH_3_-N	BOD	Volatile Phenol
Luan River	Guojiatun	↑	—	—	↓	—
Luan River	Sandaohezi	—	—	—	↓	—
Luan River	Shangbancheng	↑	↑	↑	↓	—
Luan River	Wulongji	↑	—	↑	↓	—
Yixun River	Weichang	—	—	↑	—	↑
Yixun River	Miaogong Reservoir	↑	↑	↑	↓	—
Yixun River	Longhua	↑	↓	—	—	↓
Yixun River	Hanjiaying	↑	—	↑	↓	—
Wulie River	Chengde	↑	↑	↑	↓	↑
Laoniu River	Xiabancheng	—	↓	—	—	—
Liu River	Xinglong	—	↑	↑	↓	—
Liu River	Liyingyi	↑	↓	—	—	—
Pu River	Pingquan	—	—	↑	↓	↑
Pu River	Kuancheng	—	—	—	↓	—
Sa River	Lanqiying	—	—	—	↓	—
Chao River	Dage	↑	—	↑	—	—
Chao River	Daiying	—	—	—	↓	—

Note: “↑”, “↓”, and “—” represent increase, decrease and no change, respectively.

#### 3.4.2. Attribution of Water Environment Change

**Figure 9:**
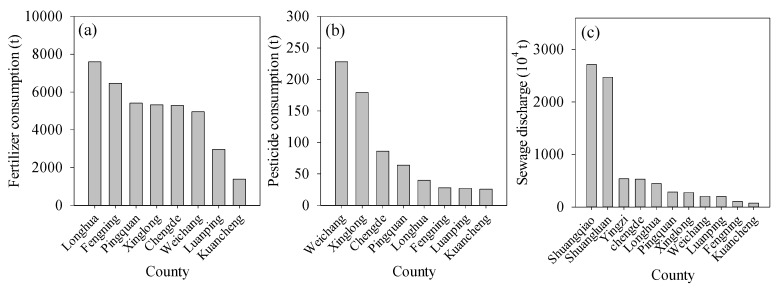
Regional distribution of (**a**) fertilizer consumption; (**b**) pesticide consumption; and (**c**) sewage discharge during 1990–2000 in Chengde.

**Figure 10:**
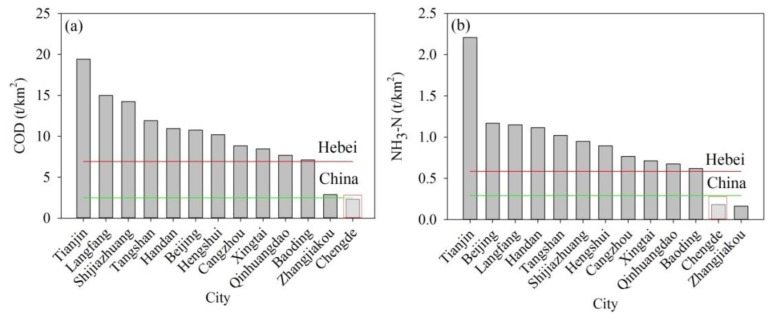
Average level of water pollutant emission in 2001–2012 in Chengde and adjacent areas: (**a**) COD; (**b**) NH_3_-H.

The changes in water pollution during the late 1980s to 2002 (Figure 6) were caused mainly by agricultural, industrial, and urban development, which caused increases in pollutant emissions. In the period 1990–2000, the total consumptions of fertilizer and pesticides in Chengde were 39,409 t and 678 t, respectively. As shown in Figure 9a,b, the largest fertilizer and pesticide consumptions occurred in Longhua and Weichang counties, with values of 7598 t and 228 t per year, respectively. Because the farmland area in these two counties is relatively large, the fertilizer and pesticide consumption in this region is greater than that in other areas. The highest values of sewage discharge were in Shuangqiao, Shuangluan, and Yingzi districts (Figure 9c). These three districts have relatively developed industry and more centralized populations, which relates to greater discharges of industrial and domestic sewage. However, since 2000, the government began to restrict waste water discharge and enhance pollutant purification treatment, which reduced water pollution to some extent. During 2001–2012, the COD and NH_3_-H emission in Chengde ranked lowest in Hebei Province (Figure 10). The average COD emission was 2.31 t/km^2^, which approached the country mean level; however, it accounted for only 33% of the Hebei Province level (Figure 10a). The NH_3_-H emission in Chengde during 2001–2012 was 0.18 t/km^2^, which accounted 31% of the Hebei Province level. However, this level is still lower than the country mean (Figure 10b). The above analysis shows that the water quality in Chengde is better than that in the adjacent areas. 

#### 3.4.3. Soil Loss (Sediment Load) Change and Attribution

**Figure 11:**
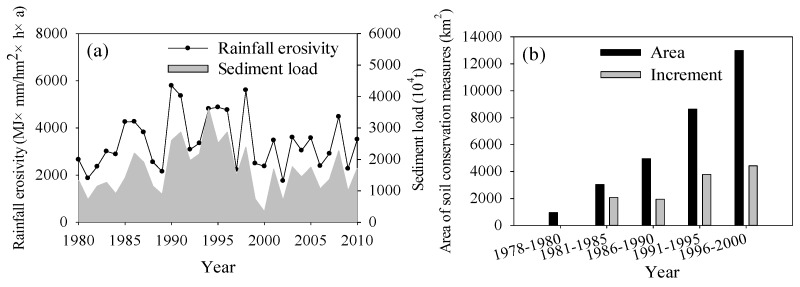
Temporal variation in (**a**) rainfall erosivity and sediment load and (**b**) the area of soil conservation measures since the late 1970s in Chengde.

In areas dominated by water erosion, rainfall erosivity and vegetation cover are the most important factors for soil loss assessment because other factors such as topography and soil property are relatively stable. Therefore, we chose these two factors for investigating the cause of soil loss change. Rainfall erosivity is an external erosion force for water erosion and thus directly influences soil erosion. During 1980–2010, the variation in rainfall erosivity and sediment load presented a consistent relationship in which rainfall erosivity weakness resulted in a sediment decrease after 1994 (Figure 11a). Soil conservation measures such as terraces and afforestation were implemented since the late 1970s, and the area reached more than 12,000 km^2^ until the end of 1990s in Chengde (Figure 11b). Therefore, soil conservation measures also played a positive role in the sediment load decline.

### 3.5. Atmospheric Environment Change and Attribution

#### 3.5.1. Atmospheric Environment Change

**Figure 12:**
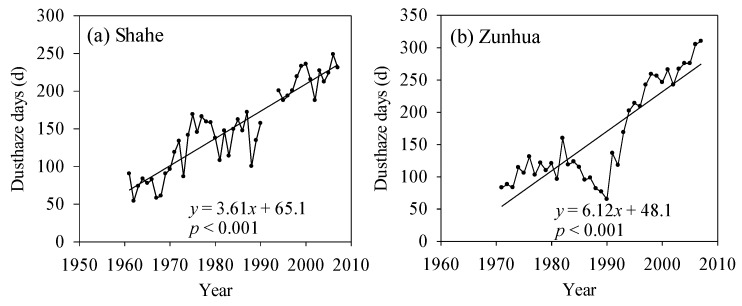
Temporal variation in dust haze days in areas adjacent to Chengde: (**a**) Shahe station in the period 1961–2007; (**b**) Zunhua station in the period 1971–2007.

Because the time span of atmospheric data is not unified and dust haze events are frequent, we discuss the changes in dust haze separately. Observation data for dust haze in Chengde were not available; therefore, we used those of Shahe and Zunhua stations in a neighboring region with similar climatic conditions. The dust haze days at Shahe station presented a clear upward trend since 1961 with a trendline slope of 3.61 days/decade (*p* < 0.001; Figure 12a). The dust haze days at Zunhua were similar to those at Shahe (Figure 12b), although a relatively low period occurred from the early 1980s to the early 1990s. This result might be attributed to the Tangshan earthquake in 1976, which blocked industrial production in Tangshan and the adjacent areas for more than 10 years; therefore, the industrial waste gas discharge was also reduced suddenly. Zunhua is a county of Tangshan and thus would have been affected by the earthquake. Since the early 1990s, industrial production has recovered gradually in this area; consequently, the dust haze days also increased. 

**Figure 13:**
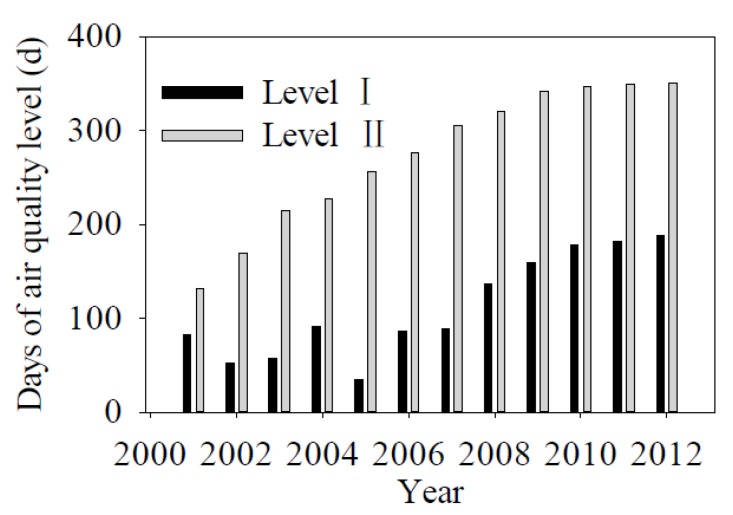
Temporal variation in days of air quality level from 2001 to 2012 in Chengde.

According to the national quality standard for air [47], from 2001 to 2012, the days of Level I air showed a clear upward trend with an increase from 83 days in 2001 to 188 days in 2012. A similar trend was noted for level II air, which increased from 132 days in 2001 to 350 days in 2012 (Figure 13).

#### 3.5.2. Attribution of Atmospheric Environment Change

**Figure 14:**
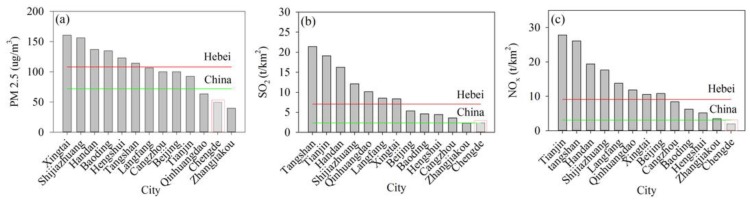
Average level of air pollutant concentrations and emissions in 2000–2012 in Chengde and adjacent areas: (**a**) PM_2.5_ concentration; (**b**) SO_2_ emission; (**c**) NO_x_ emission.

Air pollutants in Chengde originate mainly from industrial production and resident activities. Because detailed information was not available, thus we can only roughly discuss the attribution. The air pollutant concentrations and emissions in 2000–2012 in Chengde showed relatively low levels compared with those in the adjacent areas and the country or provincial mean level (Figure 14). The concentration of PM_2.5_ at Chengde station was 49.4 μg/m^3^, which is the second lowest value (Figure 14a) and 0.37 and 0.55 times the country and Hebei mean levels, respectively. The emissions of SO_2_ and NO_x_ (Figure 14b,c) showed the lowest level compared with the adjacent areas. The SO_2_ emission at Chengde station was 2.33 t/km^2^, which is close to the country mean level but only 33% of the Hebei mean level. The NO_x_ emission in Chengde was 64% and 21% of the country and Hebei mean levels, respectively. The above analysis shows that the air quality in Chengde is substantially better than that in the adjacent areas. A possible explanation is that Chengde is primarily a tourist city with little industrial waste. Although severe air pollution is not generated in the city, pollutants transported by atmospheric movement from adjacent areas can influence the air quality in Chengde.

### 3.6. Changes in Vegetation Growth and Attribution

#### 3.6.1. Changes in Vegetation Growth Status

**Figure 15:**
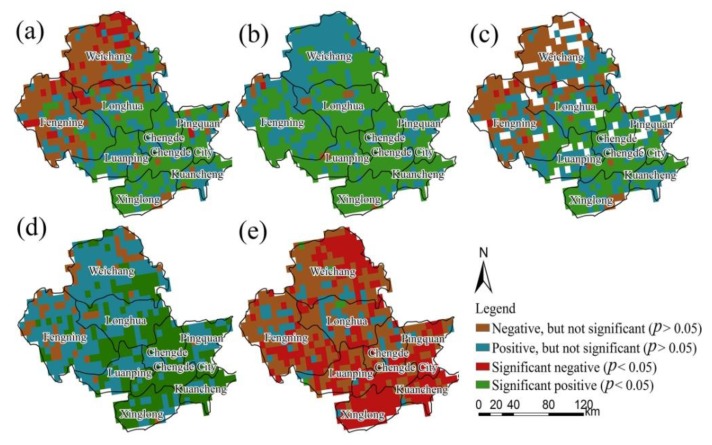
Change trends in the normalized difference vegetation index (NDVI) in Chengde during 1982–2012: (**a**) annual scale; (**b**) spring; (**c**) summer; (**d**) autumn; (**e**) winter.

The changes in the vegetation growth status during 1982–2012 showed an obvious spatial heterogeneity (Figure 15). At the annual scale (Figure 15a), the NDVI in the southeastern and central regions presented increasing trends, accounting for 67.5% of total area in Chengde; more than 50% of the area reached the significance level (*p* < 0.05). 

Moreover, the NDVI in the northwestern area including Fengning, Longhua, and Weichang a showed decreasing trend, accounting for 32.5% of the total area. However, only 10.5% passed the significance test. The overall pattern of vegetation growth change was similar to that reported in previous research [16,18] in the neighboring areas of Chengde. The slight discrepancy in the change trends and patterns can be attributed to the different research periods. For the spring (Figure 15b), the NDVI in more than 95% of Chengde showed a positive trend, with 75.8% reaching the significance level. This result indicates that vegetation activities were enhanced. The areas showing negative trends of NDVI were scattered in Longhua and Fengning counties. As shown in Figure 15c, a positive change trend in summer appeared mainly in the central and southeastern areas, and the negative trend was concentrated in the northwestern region including Fengning, Longhua, and Weichang. However, few reached the significance level. In autumn (Figure 15d), more than 90% of the area showed a positive change trend in NDVI, with 40.2% reaching the significance level. In winter (Figure 15e), more than 80% of Chengde experienced negative trends, and 49.5% passed the significance test (*p* = 0.05). This result indicates that vegetation activities were fewer in winter.

#### 3.6.2. Attribution of Vegetation Growth Status Change

Essentially, vegetation growth status is jointly influenced by climate variability and human activities. Thus, we performed correlation analysis at the pixel scale to detect significant trends in climatic parameters including annual mean temperature, annual precipitation, annual mean wind speed, and annual sunshine hours (Table 6). Correlation analysis was performed between the variation trends of climate parameters and the area (pixels) showing significant positive and negative vegetation change trends. The pixels with significant negative trends of NDVI correlated significantly with trends in annual mean temperature and annual precipitation (Table 6). All of the other correlation coefficients were not significant. A possible explanation is that Chengde is a semi-arid area of China. Precipitation was the main influencing factor for vegetation growth, and the correlation coefficient between precipitation and NDVI was larger compared with that of temperature *vs.* NDVI. It should be noted that the correlation coefficient between the annual mean temperature and NDVI with positive trends was relatively small and did not pass the significance test. A possible cause is that this area experienced large-scale ecological construction, which accelerated the vegetation restoration; thus, the vegetation growth was not controlled purely by natural conditions. However, the negative trend of NDVI was caused mainly by climate variability and declines in temperature and precipitation, particularly the latter. Since the early 1980s, a series of ecological engineering projects was implemented in Chengde and in the neighboring areas. The largest of these projects, the Grain for Green Project and the Beijing–Tianjin Sand Source Control Project, began in about 2000. As reported by Yao *et al* [48], the total area of afforestation during 2000–2006 was 153,600 ha, which is expected to play a positive role in vegetation restoration.

**Table 6:** Correlation between climate variation and significant vegetation change trends at the pixel scale in Chengde.

Change Trends of NDVI	Annual Mean Temperature	Annual Precipitation	Annual Mean Wind Speed	Annual Sunshine Hours
Significantly positive trend	0.125	0.396	−0.165	0.196
Significantly negative trend	−0.499 **	−0.633 **	−0.201	0.253

Note: ** represents *p* < 0.05 significance level.

## 4. Discussion

### 4.1. Uncertainty in the Water Resources Change Attribution

Determination of the major driving factor for streamflow changes is very important for future water resource planning and management decisions to ensure sustainable water resources utilization [49]. Most research has indicated that human activities are the major causes of runoff decrease in Haihe basin, with a contribution of more than 50% [50]. For the streamflow decrease in Luan River, Wang *et al* [51] suggested that the contribution of human activities was 57%–67% and that of climate variability was only 33%–43%. We drew similar conclusions. For changes in annual streamflow in Chengde, human activities had larger effects and contributed 66.8%; climate change contributed only 33.2%. It is important to note that a significant amount of uncertainty remains in this statistical assessment of climate and human activity on the water yield. In this study, we analyzed the reduction of streamflow and separated the effects of climatic variability and human activity. However, the values given in the tables represent average values over the baseline and the human-induced periods and do not consider the range or variability of the values. In future research, we will consider this aspect of the analysis.

### 4.2. Consistency between Local and Regional Vegetation Growth Change

**Figure 16:**
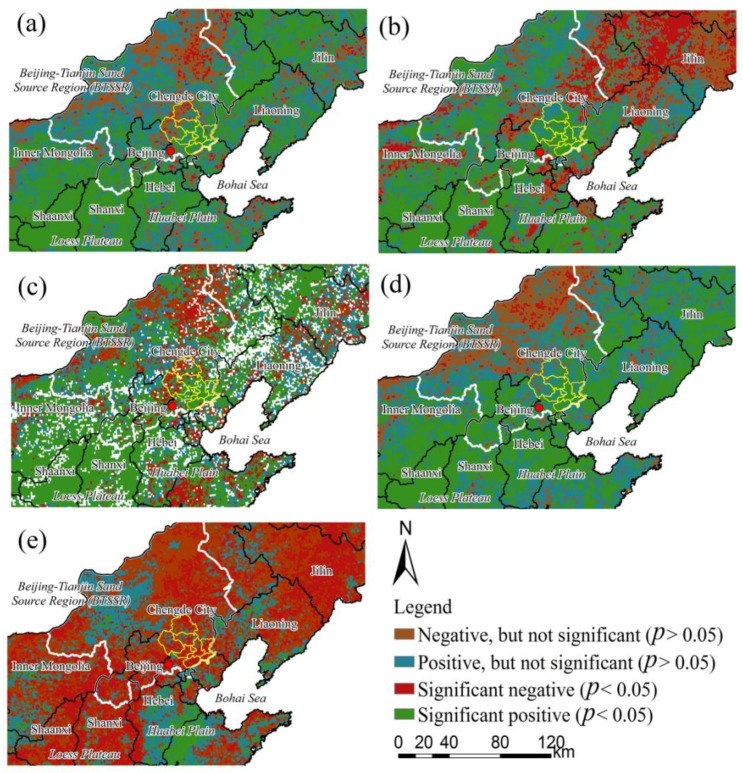
Change trends of the normalized difference vegetation index (NDVI) during 1982–2012 in Chengde and surrounding areas: (**a**) annual scale; (**b**) spring; (**c**) summer; (**d**) autumn; (**e**) winter.

The spatial patterns of trends in annual and seasonal NDVI in Chengde and the surrounding areas for the period 1982–2012 are shown in Figure 16. A high degree of spatial heterogeneity was detected that varied annually and seasonally. At the annual scale (Figure 16a), the NDVI trends were positive in most areas, particularly in northern Shaanxi and Shanxi and in Hebei Province including central and southeastern Chengde. However, decreasing trends were noted in the small sandy area of southern Horqin and in adjacent areas including Weichang, Fengning, and Longhua counties. 

In the spring (Figure 16b), NDVI trends were positive in most areas of the *Beijing–Tianjin Sand Source Region* (BTSSR), particularly in Chengde. However, northern Liaoning, Jilin, and northeastern Inner Mongolia showed decreasing trends. The summer NDVI showed a fragmented pattern (Figure 16c) including increases in most areas of Chengde and in northern Shaanxi and Shanxi. However, some regions experienced a decrease in summer NDVI, such as the desert grassland, southern Greater Hinggan, and the sandy area of southern Horqin, which lies in Inner Mongolia. Most areas in Chengde and provinces other than Inner Mongolia experienced a positive trend of autumn NDVI during 1982–2012 (Figure 16d). The decreasing regions were included a southwest-to-northeast band in the BTSSR. In winter (Figure 16e), the NDVI presented decreasing trends in most areas except for eastern Hebei Province, the central part of the BTSSR, and other fragmented areas. 

The spatial patterns of annual and seasonal vegetation growth changes shown in Figure 16 were similar to those previously reported [52,53,54] during the same period. Liu *et al* [52] investigated vegetation cover changes in China from 1982 to 2012 by using integrated GIMMS NDVI and Moderate Resolution Imaging Spectroradiometer (MODIS) NDVI datasets. They reported significant increases in the NDVI at the national scale (*p* < 0.05) during past three decades, particularly in northern China. The two distinct periods were 1982–1997 and 1997–2012, with slopes of 1.2% and 0.6% per decade, respectively. Qu *et al.* [53] used GIMMS NDVI3g datasets from 1982 to 2011 and linear trend analysis to monitor vegetation activity in China during the growing season between the onset and end of plant growth. They reported significant positive trends in some areas in northern China including Chengde (*p* < 0.05), which experienced human-induced impacts. The intensive changes occurred in densely populated and afforestation areas. Zhao *et al.* [54] also found increasing trends in greenness in the Three North region of China during 1982–2000 and 2000–2013, particularly in the latter period, by using a linear model and Mann–Kendall trend detection. Although small differences appeared in the comparison of similar studies, the spatial patterns of vegetation change and trend are reliable because the data source, processing method, and time span of dataset differed.

## 5. Implications of Environmental Change and Ecological Construction

Considering the intensive climate variability, human activities, and ecological conservation, Chengde has great significance for investigating regional environment changes under a complicated background. The water resources and water environment are of high importance, particularly streamflow reduction and water pollution in the main rivers. Thus, adequate planning is necessary to actively respond and adapt to the possible effects of future changes in climate, population growth, and industrial development to ensure sustainable development and ecological safety. In particular, the impacts of climatic and anthropogenic factors on water resources, the water environment, and the atmospheric environment should be closely monitored. Moreover, the current ecological restoration reflected by NDVI increases is the cumulative effect of ecological projects and climate variability; however, the current restoration is only local and temporary and does not represent overall or fundamental improvement. The current measures undertaken in ecological projects, such as tree and grass planting, are relatively simple and rarely include soil remediation and the control of air and water pollution. The public awareness of water conservation and environmental protection should be enhanced in the future through education. Rapid population growth and industrial development can be expected in future decades; thus, environment managers should pay particular attention to the environmental impacts of such factors.

## 6. Conclusions

This study quantitatively assessed the environmental changes in Chengde city on the basis of observation records of water resources, water environment, atmospheric environment, and vegetation activity and investigated the possible causes. The main conclusions are summarized in the following points:

(1) In Chengde, the annual mean temperature increased significantly from 1956 to 2011, and the annual precipitation fluctuated severely. The most humid period, 1970–1980, was followed by the driest period in 1980–1990.
(2) The land cover change in 2000–2010 was not obvious. In comparison with 1985 and 2010, the main changes occurred in woodland, farmland, and residential areas, which were caused jointly by ecological projects, urbanization, and population growth.
(3) The streamflow in Chengde presented a downward trend from the late 1950s to 2002, with an abrupt change occurring in 1979. Streamflow reduction was induced jointly by climate variability and human activities, with contributions of 33.2% and 66.8%, respectively.
(4) In the period 2001–2012, clear upward trends were shown in the days of levels I and II air quality. Moreover, the air pollutant concentrations and emissions in Chengde showed relatively low levels compared with those in the adjacent areas and countries or provincial mean levels. That is, the air quality improvement was significantly greater than that in the adjacent areas. The air quality changes were closely related to pollutant emissions induced by anthropogenic activities.
(5) During 1993–2000, the pollutant of MnO^4−^ and volatile phenols at most stations maintained stability. Hardness and NH_3_-N at more than 50% stations showed upward trends, and the BOD at 70.6% stations showed downward trends, which is a sign of water quality deterioration. The water quality began to improve after 2002, and the water quality above level III at monitoring stations increased significantly. The water quality changes were closely related to pollutant emissions induced by anthropogenic activities.
(6) The changes in vegetation growth in Chengde during 1982–2012 showed obvious spatial heterogeneity. At the annual scale, vegetation in the southeastern and central regions presented restoration trends, and the vegetation in the northwestern area showed a degradation trend. The pixels with obvious degradation trends correlated significantly with the annual mean temperature and annual precipitation. Ecological engineering also played a positive role in the vegetation restoration.

## Supplementary Materials

The following are available online at www.mdpi.com/1660-4601/13/2/165/s1, Table S1: Summary of hydrological characteristics in the Chengde city and neighboring basins, Table S2: Summary of meteorological stations in the Chengde city and neighboring, Table S3: Summary of water quality monitoring stations in the Chengde city and neighboring area, Table S4: Summary of air quality monitoring stations in the Chengde city and neighboring area.
