# Supplementary material for: Environmental Change in the Agro-Pastoral Transitional Zone, Northern China: Patterns, Drivers, and Implications
Source: Int J Environ Res Public Health. 2016 Jan 28;13(2):165. doi: 10.3390/ijerph13020165 (PMC4772185; doi:10.3390/ijerph13020165)
Supplement: Supplementary file 1 [file ijerph-13-00165-s001.pdf]

Supplementary Materials: Environmental Change in the Agro-pastoral Transitional Zone, Northern China: Patterns, Drivers, and Implications

Chong Jiang and Fei Wang

Table S1. Summary of hydrological characteristics in the Chengde city and neighboring basins.

ID	River	Gauge Station	River Length (km)	Drainage Area (km^2)	Location		Time Series
					Latitude (° ′ N)	Longitude (° ′ E)	
1	Luan River	Sandaohezi	522.8	17,100	41°02′	118°19′	1958–2002
2	Xingzhou River	Boluonuo	84.5	1378	41°04′	117°20′	1960–2002
3	Wulie River	Chengde	99.0	2200	40°46′	118°10′	1957–2002
4	Liu River	Liying	63.5	626	41°25′	116°35′	1957–2002
5	Yixun River	Hanjiaying	217.4	6787	41°48′	117°19′	1957–2002
6	Laoniu River	Xiabancheng	69.5	1615	40°45′	118°10′	1968–2002

Table S2. Summary of meteorological stations in the Chengde city and neighboring

ID	Gauge Station	Location		Time Series
		Latitude (° ′ N)	Longitude (° ′ E)	
1	Yebaishou	41°23′	119°42′	1956–2011
2	Qinhuangdao	39°51′	119°31′	1956–2011
3	Huailai	40°24′	115°30′	1956–2011
4	Zhangjiakou	40°47′	114°53′	1956–2011
5	Chifeng	42°16′	118°56′	1956–2011
6	Qinglong	40°24′	118°57′	1956–2011
7	Zunhua	40°12′	117°57′	1956–2011
8	Miyun	40°23′	116°52′	1956–2011
9	Duolun	42°11′	116°28′	1956–2011
10	Fengning	41°13′	116°38′	1956–2011
11	Weichang	41°56′	117°45′	1956–2011
12	Chengde	40°59′	117°57′	1956–2011

Table S3. Summary of water quality monitoring stations in the Chengde city and neighboring area.

ID	River	Station	Location		Time Series
			Latitude (° ′ N)	Longitude (° ′ E)	
1	Luan River	Guojiatun	41°34′	117°06′	1993–2000
2	Luan River	Sandaohezi	41°02′	118°19′	1993–2000
3	Luan River	Shangbancheng	40°49′	118°03′	1987–2001
4	Luan River	Wulongji	40°42′	118°08′	1987–2001
5	Yixun River	Weichang	41°56′	117°45′	1993–2000
6	Yixun River	Miaogong Reservior	41°45′	117°49′	1993–2000
7	Yixun River	Longhua	41°18′	117°44′	1993–2000
8	Yixun River	Hanjiaying	41°48′	117°19′	1993–2000
9	Wulie River	Chengde	40°46′	118°10′	1993–2000
10	Laoniu River	Xiabancheng	40°45′	118°10′	1993–2000
11	Liu River	Xinglong	40°25′	117°30′	1990–2001
12	Liu River	Liyingyi	41°06′	117°59′	1993–2000
13	Pu River	Pingquan	41°01′	118°42′	1990–2001
14	Pu River	Kuancheng	40°36′	118°29′	1993–2000
15	Sa River	Lanqiying	40°23′	118°01′	1993–2000
16	Chao River	Dage	41°12′	116°38′	1985–2001
17	Chao River	Daiying	40°42′	117°10′	1993–2000

Table S4. Summary of air quality monitoring stations in the Chengde city and neighboring area.

ID	Station	Location		Time Series
		Latitude (° ′ N)	Longitude (° ′ E)	
1	Xingtai	37°04′	114°30′	2000–2012
2	Shijiazhuang	38°02′	114°30′	2000–2012
3	Handan	36°37′	114°32′	2000–2012
4	Baoding	38°52′	115°27′	2000–2012
5	Hengshui	37°44′	115°40′	2000–2012
6	Tangshan	39°37′	118°10′	2000–2012
7	Langfang	39°32′	116°41′	2000–2012
8	Cangzhou	38°18′	116°50′	2000–2012
9	Beijing	39°54′	116°24′	2000–2012
10	Tianjin	39°05′	117°12′	2000–2012
11	Qinhuangdao	39°56′	119°36′	2000–2012
12	Chengde	40°57′	117°57′	2000–2012
13	Zhangjiakou	40°46′	114°53′	2000–2012

© 2016 by the authors; licensee MDPI, Basel, Switzerland. This article is an open access article distributed under the terms and conditions of the Creative Commons by Attribution (CC-BY) license (http://creativecommons.org/licenses/by/4.0/).
